# Advances and Challenges in Breast Cancer Management: A History Made of Evolutions and Revolutions

**DOI:** 10.3390/cancers15061713

**Published:** 2023-03-10

**Authors:** Yazid Belkacemi

**Affiliations:** Department of Radiation Oncology and Henri Mondor Breast Center, Henri Mondor University Hospital, (APHP), University of Paris Est Créteil (UPEC), INSERM Unit 955 (i-Bio), Institut Mondor dec Recherche Biomédicale (IMRB), 94010 Creteil, France; yazid.belkacemi@aphp.fr; Tel.: +33-1-49-81-45-22; Fax: +33-1-49-81-25-89

Jean Jaurès (1859–1914) stated that “Human history is but a ceaseless effort of invention, and perpetual evolution and creation”. The keywords of this quote, “invention”, “evolution” and “creation”, find an extraordinary echo in the unique history of Oncology.

The fascinating story of Hippocrates’ “crab” (460–377 BC) has evolved at the pace of clinical descriptions, bio-pathological discoveries, the validation of new therapeutic molecules, technological developments and links forged with other specialties based on multidisciplinary activities.

The other beautiful story to tell is that of research in the fight against cancer, which consists of a mixture of explorations, trial and error and breakthroughs, thanks to inventions, sometimes from necessity, transforming the story into real revolutions,… “necessity is the mother of invention”.

## 1. Benchmarks and Tools in Cancer Care History

For solid tumors, surgery has long been an unavoidable weapon. The complete removal of a tumor has been and still is, in many situations, a guarantee of long-term disease control. The first descriptions date back to 1600 BC. Due to a great many advances, such as the development of anesthesia (1846) and antisepsis (1867), cancer surgery has developed into its current form. During the 19th and 20th centuries, surgical techniques developed for specific organs, with the first being laryngectomy (1873), followed by gastrectomy (1881), then Halsted-type mastectomy (1890), prostatectomy (1904) and hysterectomy (1906) [1].

This period was also extraordinarily full of history for other locoregional treatments, such as radiotherapy. After Roentgen discovered X-rays (1895), the speed of the discovery of other ionizing radiation types, especially regarding their application to the treatment of tumors, was dizzying. From 1896, with the treatment of stomach cancer (by Victor Despeignes in 1896) until the middle of the 1900’s, radium, discovered by Marie Curie in 1898, was used in several neoplasms forms before the discovery of cobalt and cesium. The story then continued with major turning points, such as the use of linear gas pedals (1940), the use of scanners for 3D treatment planning (1971), the use of multimodal imaging for target definition (1990s) and, finally, on-board imaging and intensity modulation (2000s) [2]. To illustrate the latest advances, this Special Issue is devoted to articles focusing on “innovations and new concepts in radiation oncology”.

## 2. Concepts and Strategies in Breast Cancer Care History

As early as 1889, Stephen Paget described the “seed and soil” theory, according to which metastases are not randomly distributed in the organism but according to an appropriate microenvironment [3]. Thus, cancer cells would produce cytokines and growth factors that would activate the recruitment/”homing” and mobilization of medullary endothelial progenitors necessary for neovascularization and the growth of metastases in what were considered pre-metastatic niches, themselves under the governance of messengers of the primary tumor [4]. Thus, as knowledge of metastatic disease improved, the challenge, which at the beginning of the 20th century was focused on local treatments, was now to integrate sequential combinations of local with systemic treatments (in adjuvant situation) or the early initiation of combined neoadjuvant therapy to increase the chances of organ preservation and reduce the frequency of systematic, mutilating radical surgery. In these combined regimens, chemotherapy rapidly gained acceptance in multiple locations to reduce the initial tumor volume and treat micro-metastatic disease through spatial cooperation [5,6]. Thus, the drug combinations that were initially developed to ensure the concept of “maximum tolerable treatment” were progressively revisited thanks to therapeutic personalization, leaving room for “minimal effective treatment” in breast cancer [7]. This development was made possible by countless innovations in Medical Oncology, whose new concepts and new systemic molecules are the subject of two separate articles in this Special Issue.

## 3. New Therapies in Breast Cancer Care

The development of new predictive markers of treatment efficacy has revolutionized practice and sometimes led to spectacular results never obtained before, as seen in the individualization of treatments. The duration, type and sequence of treatments have been “revisited” thanks to this possibility of the better therapy personalization. Historically, endocrine therapies were among the first targeted treatments to be based on predictive factors of response, particularly in breast cancer, with estrogen and progesterone receptors. In hormone-dependent cancers, therapeutic de-escalation (as opposed to multidrug therapy) has developed spectacularly, with a significant improvement in the quality of life of patients without prejudice regarding oncological outcomes [8]. Moreover, among the major developments in terms of targeting therapies in the 2000s were the targeting of the HER2 oncoprotein (for breast and gastric cancers) and angiogenesis (for gastro-intestinal and lung cancers). This new family of targeted therapies has made it possible to achieve unprecedented survival rates by using data obtained in the laboratory several years before the transition to the clinic in selected patient subpopulations [9]. For the concurrent administration of anti-HER2 drugs and radiation, early data indicated safety with the prerequisite of reducing as low as possible heart exposure, particularly in the case of internal mammary chain irradiation [10,11]. In this Special Issue, new hormone and targeted therapies will be specifically discussed.

Another area in which chemotherapy has been “supplanted” by innovative therapy is immunotherapy. Immunomodulation is a well-known concept in onco–hematology and has been used for decades for patients receiving ablative conditioning therapy before marrow or peripheral stem cell transplantation. Recent developments in immunity targeted therapies have allowed a progressive extension to a growing number of tumor locations, such as triple-negative breast cancer [12].

Increasingly detailed knowledge concerning tumor biopathology has made it possible to define subgroups of patients who, due to these molecules, have seen their disease become chronic. Thus, over the past 12 years, immune checkpoint inhibitors used either alone or in combination with other systemic therapies have generated considerable progress in metastatic and adjuvant settings of breast cancer. At the same time, new and unexpected questions have arisen regarding the management of their long-term toxicities and the strategy to adopt for responders throughout these treatments or during concurrent radiotherapy delivery [13]. In this Special Issue, these advances will be addressed in terms of the cancers in which the results have been most significant.

## 4. Patients Age in Oncology

In 2023, a Special Issue concerning Oncology cannot be produced without devoting a chapter to oncogeriatric assessment and its integration into multidisciplinary therapeutic decisions. Oncogeriatric assessment has become an essential step in the treatment of a large number of elderly patients at all stages of their cancer. Adapting treatments to the geriatric status and frailties of patients has become a standard in the management of cancers in the elderly.

Epidemiological data from industrialized countries show an increasing incidence of cancers in increasingly elderly populations, leaving no alternative to the best personalization of treatments, taking into account their effectiveness and their impact on quality of life. The practical organization of treatment paths has been largely anticipated for several years by the development of very close collaboration between both specialties’ oncology and geriatric. The integration of these exchanges in continuing education programs, the use of simple tools for an initial geriatric assessment (such as the G8 scale) or the use of more in-depth assessment scales and the development of multidisciplinary meetings that systematically include both specialties in the discussion, have greatly facilitated the process and have made it possible to create recommendations and protocols adapted to elderly patients for whom the personalization of systemic treatments, de-escalated protocols and the adaptation of hypofractionated radiotherapy regimens are required more than ever for a better benefit/risk ratio [14]. In this Special Issue, an entire article will be devoted to the contribution of oncogeriatrics regarding the management of elderly breast cancer patients, which will undoubtedly be a major epidemiological problem in the coming decades for Oncology.

## 5. Conclusions

The recent advances in radiotherapy practice and the development of new drugs and diagnostic tools in Oncology are a real revolution in breast cancer management following the multimodality strategies. These advances have allowed reduced sequelae rates with concomitantly better local control and improved survival and quality of life for many patients. The current concepts using treatment personalization have become a reality, with better tumor targeting and systematic biopathology used to predict the efficacy of the combined strategies sequenced to reach the best benefit/risk ratio. In adjuvant and metastatic breast cancer settings, the opportunities for the concurrent administration of new drugs with radiotherapy are increasing. It is essential to pay great attention to the risks incurred by these associations, as the data are not sufficient to draw strong guidelines. This Special Issue is an excellent opportunity to discuss optimal management today and the prospects for tomorrow, considering the perpetual evolutions in the study of breast cancer.

## References

[1] https://fr.wikipedia.org/wiki/Histoire_de_la_m%C3%A9decine.

[2] Gérard J.P., Thariat J., Giraud P., Cosset J.-M. (2010). Past, present and near future of techniques in radiation oncology. Bull. Cancer.

[3] Poste G., Paruch L. (1989). Stephen Paget, M.D., F.R.C.S., (1855–1926) A retrospective. Cancer Metastasis Rev..

[4] Sugarbaker E.V. (1979). Cancer metastasis: A product of tumor-host interactions. Curr. Probl. Cancer.

[5] Brady L.W., Markoe A.M., Fisher S.A., Micaily B. (1988). Cancer cure with organ preservation using radiation therapy. Acta Oncol..

[6] Trimble E.L., Ungerleider R.S., Abrams J.A., Kaplan R.S., Feigal E.G., Smith M.A., Carter C.L., Friedman M.A. (1993). Neoadjuvant therapy in cancer treatment. Cancer.

[7] Veronesi U., Stafyla V., Veronesi P. (2012). Breast cancer: From “maximum tolerable” to “minimum effective” treatment. Front. Oncol..

[8] Weiser R., Polychronopoulou E., Kuo Y.F., Haque W., Hatch S.S., Tyler D.S., Gradishar W.J., Klimberg V.S. (2021). De-escalation of endocrine therapy in early hormone receptor-positive breast cancer: When is local treatment enough?. Ann. Surg..

[9] Hortobagyi G.N. (2005). Trastuzumab in the treatment of breast cancer. N. Engl. J. Med..

[10] Belkacémi Y., Gligorov J., Ozsahin M., Marsiglia H., De Lafontan B., Laharie-Mineur H., Aimard L., Antoine E.-C., Cutuli B., Namer M. (2008). Concurrent trastuzumab with adjuvant radiotherapy in HER2-positive breast cancer patients: Acute toxicity analyses from the French multicentric study. Ann. Oncol..

[11] Belkacemi Y., Gligorov J. (2010). Concurrent trastuzumab—Internal mammary irradiation for HER2 positive breast cancer: “It hurts to be on the cutting edge”. Radiother. Oncol..

[12] Sugie T., Salgado R., Fong L. (2022). Advancements in immunology and immunotherapy for breast cancer. Front. Oncol..

[13] Haanen J., Obeid M., Spain L., Carbonnel F., Wang Y., Robert C., Lyon A., Wick W., Kostine M., Peters S. (2022). Management of toxicities from immunotherapy: ESMO Clinical Practice Guideline for diagnosis, treatment and follow-up. Ann. Oncol..

[14] Levassort H., Pépin M., Teillet L., Ghebriou D., Cudennec T. (2022). Oncogeriatric assessment: The first step in personalizing cancer treatment in the elderly. Rev. Med. Interne.

